# Composite Materials from Renewable Resources as Sustainable Corrosion Protection Coatings

**DOI:** 10.3390/polym13213792

**Published:** 2021-11-02

**Authors:** Raluca Sanda Komartin, Brindusa Balanuca, Madalina Ioana Necolau, Anca Cojocaru, Raluca Stan

**Affiliations:** 1 Department of Organic Chemistry “C. Nenitescu”, Faculty of Applied Chemistry and Materials Science, University Politehnica of Bucharest, 1-7 Polizu Street, 011061 Bucharest, Romania; ralu.komartin@gmail.com (R.S.K.); brindusa.balanuca@yahoo.com (B.B.); 2 Advanced Polymer Materials Group, University Politehnica of Bucharest, 1-7 Polizu Street, 011061 Bucharest, Romania; madalinanecolau@gmail.com; 3 Department of Inorganic Chemistry, Physical Chemistry and Electrochemistry, Faculty of Applied Chemistry and Materials Science, University Politehnica of Bucharest, 1-7 Polizu Street, 011061 Bucharest, Romania; anca.cojocaru@upb.ro

**Keywords:** epoxidized linseed oil, lignin, composites, anti-corrosion coating

## Abstract

Epoxidized linseed oil (ELO) and kraft lignin (LnK) were used to obtain new sustainable composites as corrosion protection layers through a double-curing procedure involving UV radiation and thermal curing to ensure homogeneous distribution of the filler. The crosslinked structures were confirmed by Fourier-transform infrared spectrometry (FTIR), by comparative monitorization of the absorption band at 825 cm^−1^, attributed to the stretching vibration of epoxy rings. Thermal degradation behavior under N2 gas indicates that the higher LnK content, the better thermal stability of the composites (over 30 °C of Td10% for ELO + 15% LnK), while for the experiment under air-oxidant atmosphere, the lower LnK content (5%) conducted to the more thermo-stable material. Dynamic-mechanic behavior and water affinity of the new materials were also investigated. The increase of the Tg values with the increase of the LnK content (20 °C for the composite with 15% LnK) denote the reinforcement effect of the LnK, while the surface and bulk water affinity were not dramatically affected. All the obtained composites were tested as carbon steel corrosion protection coatings, resulting in significant increase of corrosion inhibition efficiency (IE) of 140–380%, highlighting the great potential of the bio-based ELO-LnK composites as a future perspective for industrial application.

## 1. Introduction

Polymer composites are widely used in the fields of engineering due to their performance and versatility, making them adaptable in different industrial sectors. In recent years, numerous studies have been developed for the progress of the bio-based epoxy derivatives, to face out the use of conventional epoxy resins and to achieve great industrial gains [1,2].

Due to their advantages of natural origin, structure versatility, and worldwide availability, vegetable oils (VO) were considered as high-value raw materials in the synthesis of epoxy derivatives (epoxidized vegetable oil–EVO), but there are several drawbacks related to the EVO-based polymeric matrices (e.g., inadequate thermo-mechanical properties or poor mechanical resistance). Many attempts were conducted to solve these issues through the development of different or sophisticated curing procedures or by using reinforcing agents or fillers. For example, mixing different types of conventional epoxy resins with EVO, special design of the crosslinking agents or employing modified vegetable oil structures as crosslinking agents, were reported [2,3].

Literature data present various methods to augment the performances of the conventional resins, using natural curing and co-curing agents as acids, anhydrides, amines, and their derivatives, or natural reinforcing agents like modified cellulose, natural fibers, or powder inorganic fillers [4,5,6]. One of the important applications related to the epoxy-based coatings is anticorrosion protection, their properties being extensively investigated due to their great advantages in terms of mechanical properties, thermal or chemical stability, and high efficiency upon corrosion inhibition [7,8,9]. Even if most of them are conventional materials, the frequency of using EVO-based counterparts is continuously growing, different EVO types being successfully used to produce anticorrosion coatings, due to the availability as starting materials, increased hydrophobicity of the resulted layers or convenient reactivity of the epoxy groups [10,11,12].

Recently, lignin was investigated as potential filler for different anticorrosion coatings, demonstrating a promising inhibition potential in aggressive media [13,14,15]. Moreover, lignin represents the second natural organic material in terms of occurrence and recent technological development made it available on industrial scale employing industrial waste. Major advantages associated with lignin such as biodegradability, CO_2_ neutral, antioxidant, antimicrobial, and stabilizer properties added to the possibility of chemical functionalization are opening new perspectives and a whole new chemistry for applications, association of functionalized lignin’s with conventional epoxy resins being recently reported [16].

With the aim to reduce the use of non-renewable resources, this paper provides an investigation of bio-based composite materials obtained from epoxidized linseed oil (ELO) and kraft lignin (LnK) as natural filler, and an investigation of the anticorrosion performance of the obtained coatings in aggressive media. The designed materials’ (ELO-LnK) fabrication involved a double-curing procedure to overcome the inconvenience of lignin sedimentation: first, a short-time UV treatment “to catch” the lignin within continuous oil matrix and then a long-term thermal curing to obtain proper polymeric composites with superior yields for the ring-opening reaction in the presence of a commercial crosslinking system. The ELO-LnK composites were investigated by Fourier transform infrared spectrometry (FTIR), thermo-gravimetric analysis (TGA), dynamic mechanical analysis (DMA), contact angle measurements (CA), water absorption degree (WAD), and scanning electron microscopy (SEM). The obtained bio-based epoxy-lignin coatings mitigate the corrosion processes for the carbon steel and a significant improvement of the corrosion inhibition efficiency is observed.

The novelty of the research resides both in association of the two bio-based starting materials, epoxidized linseed oil and kraft lignin, for the synthesis of the new composites and a dual-curing procedure using UV radiation and thermal treatment for hardening the oil-based resin. Thus, a major drawback regarding lignin sedimentation was surpassed reaching adequate features for the studied composite materials in terms of thermal and thermo-mechanical performances and their anticorrosion properties.

## 2. Materials and Methods

### 2.1. Materials

Linseed oil (LO) obtained by cold-pressing process was acquired from PTG Deutschland, Flurstedt, Germany. Kraft lignin (LnK), the photo-initiator triarylsulfonium hexafluoro-antimonate (THA) and the solvents and reagents used for the epoxidation of LO were purchased from Sigma-Aldrich (subsidiary of Merck KGaA, Darmstadt, Germany). The dodecenyl succinic anhydride hardener (Araldite HY964, HY) and the 2,4,6-tris(dimethylaminomethyl)phenol accelerator (Araldite DY064, DY) were provided by Merck. (Darmstadt, Germany). The products were used as received.

### 2.2. Methods

#### 2.2.1. Synthesis of ELO

The epoxidation of the double bonds on the fatty acid structures contained by LO was performed according to procedures described in the literature for LO and other vegetable oils using peracetic acid generated in situ [17,18,19,20,21]. Briefly, the synthesis protocol involves the conversion of the double bonds into epoxy rings using H_2_O_2_ and glacial acetic acid (H_2_O_2_/acetic acid/unsaturation molar ratio: 10/2/1), in the presence of H_2_SO_4_ (50% solution) as reaction catalyst and toluene as reaction solvent and diluent of the oily phase. After 22 h at 65 °C, under constant magnetic stirring, the reaction was stopped and the product was purified. The purified ELO product (reaction yield~94%) was structurally characterized and used as continuous phase for the composite’s fabrication.

#### 2.2.2. Preparation of the ELO_LnK Composite Coatings

A certain amount of ELO and HY964, calculated based on epoxy rings:anhydride molar ratio (2:1), was mixed until visual homogenization. Varying amounts of LnK (5%—sample S1, 10%—sample S2, and 15%—sample S3, respectively, calculated as based on the amount of ELO) were added and homogenized using a Bandelin Sonorex sonication bath for 15 min. The accelerator DY064 (5% wt. with respect to ELO) and the photo-initiator THA (4% wt. against ELO) were then added, manually blended and then sonicated for 5 min [22,23]. A reference sample (Sr) without LnK content was formulated using the same procedure.

To obtain protective layers, carbon steel sheets (15 mm wide × 35 mm long × 0.5 mm thick) were used as substrate for the oil-based coating. Prior to the deposition, the metal sheets were pickled by immersion in HCl solution (10%), then washed with distilled water and finally with isopropanol. The ELO-LnK mixtures (S1–S3 and Sr) were applied on the metal surfaces as uniform and thin films and then subjected to the dual-curing process, with UV irradiation for 15 min (λ = 365 nm, power 8 W, maintaining a distance of 10 cm between radiation source and sample surface) and thermal treatment at 80 °C for 23 h. The samples coated with ELO-LnK were tested for anticorrosion performance.

The obtained coated steel plates were tested to define the potential of ELO-LnK composites as anticorrosion protection layers. Likewise, the obtained mixtures were slowly poured in the manufactured silicone molds (20 mm wide × 35 mm long, with respect for the thickness of the layers made on the metal plates, about 0.5 mm) and subjected to the same dual-curing process. The obtained composite materials were subjected to different characterization techniques to establish their general material features.

### 2.3. Characterization

#### 2.3.1. Nuclear Magnetic Resonance Spectroscopy (NMR)

^1^H-NMR spectra were recorded on a Bruker Advance III Ultrashield Plus 500 MHz spectrometer (Billerica, MA, USA), operating at 11.74 T, corresponding to the resonance frequency of 500.13 MHz for the ^1^H nucleus. Chemical shifts are reported in ppm, using TMS as internal standard.

#### 2.3.2. Gel Fraction Measurements (GF)

Tetrahydrofuran extraction (24 h) was made for the obtained ELO-based materials (weight *w*1), to determine the soluble fraction. After extraction, materials were dried to constant weight (60 °C; weight *w*2). All measurements were performed in triplicate. *GF* was calculated using the equation:

(1) $$GF=\frac{\left(w1-w2\right)}{w1}\times 100$$

#### 2.3.3. Fourier Transform Infrared Spectrometry (FTIR)

FTIR spectra were registered on a Vertex 70 Brucker FTIR spectrometer equipped with an attenuated total reflectance (ATR), at room temperature. Runs with 32 scans in 600–4000 cm^−1^ wave number region were used.

#### 2.3.4. Thermo-Gravimetric Analysis (TGA)

Thermal stability and decomposition profile of the synthesized materials were studied by TGA in both inert and aerobic atmosphere, using a Q500 TA instrument. Thermal degradation was investigated for all samples in the temperature range 25–700 °C, at a heating rate of 10 °C/min under nitrogen/air flow (90 mL/min).

#### 2.3.5. Dynamic Mechanical Analysis (DMA)

Thermo-mechanical features of the ELO-LnK composites were measured on a Tritec 2000 instrument, operated in single cantilever bending mode. Rectangular materials (20 mm long × 10 mm wide × 5 mm thick) were tested at 1 Hz frequency, ramping the temperature from −60 to 100 °C (5 °C/min heating rate).

#### 2.3.6. Contact Angle Measurements (CA) and Water Absorption Degree (WAD)

Static CA values were measured for the composite materials at room temperature on DSA100E (KRUSS GMBH) equipment using Drop shape analysis method. Ultrapure water droplets were used with a drop volume of approximately 2 μL. CA values were measured within 10 s of the drop contacts with the surface. Reported values represent the average of three determinations for each specimen. Additionally, WAD was determined in triplicate, using the standard water absorption ASTM D570 method.

#### 2.3.7. Scanning Electron Microscopy (SEM)

Composites morphology was explored by scanning electron microscopy, using cross-sections. The samples were manually broken in liquid nitrogen, sputtered with a thin layer of gold, and scanned using a Quanta Inspect F SEM device equipped with a field emission gun with a resolution of 1.2 nm.

#### 2.3.8. Electrochemical Measurements

Potentiodynamic polarization measurements and electrochemical impedance spectroscopy (EIS) were performed using a Potentiostat/Galvanostat Voltalab 40 (Radiometer Analytical). Potentiodynamic polarization experiments were performed at a sweep rate of 2.5 mV/s, in the potential range from −0.8 V/SSCS to +0.8 V/SSCS. EIS studies were performed in a frequency range 100 kHz–50 mHz using a sinusoidal perturbation with a voltage amplitude of 10 mV. The impedance spectra were plotted both before the potentiodynamic polarization test in the aggressive environment (after 30 min immersion) and after the accelerated corrosion test. Using EIS data, the electrochemical parameters were calculated, namely the charge transfer resistance (*R_ct_*) and the double layer capacitance (C_dl_). The electrochemical cell was a thermostated three-electrode one and the experiments were performed in 3.5% NaCl media. The working electrode consisted of the carbon steel samples (wt.%, C: 0.049, Mn: 0.227, Cr: 2.34, S: 0.0005, Fe: rest) coated with the anticorrosive coatings tested (active surface aria 0.5 cm^2^), the auxiliary electrode was a platinum electrode (4.5 cm^2^ active surface), and the reference electrode a Ag/AgCl/KCl sat. electrode from Radiometer Analytical (SSCS) immersed into the working solution. All potential values were referred to this reference electrode. All experiments were conducted with naturally aerated solutions at 25 ± 1 °C. Prior to each experiment, the working electrode was immersed in a 3.5% NaCl solution for 30 min to attain a quasi-stationary state.

## 3. Results and Discussion

### 3.1. ELO Characterization

The epoxidation reaction of the LO was monitored by ^1^H-RMN and FTIR. Structural changes were identified in the ^1^H-NMR spectrum recorded for ELO as compared to the spectrum registered for the crude oil. For ELO compound (Supplementary Material, Figure S1b), new signals at 3.02 and 2.85 ppm, specific to internal and marginal protons belonging to epoxy rings and respectively 1.60 ppm, assigned to the protons from the –CH_2_ groups located between two epoxy rings (originated from linolenic and linoleic acids) were observed. The signal at 5.25 ppm, attributed to the protons of the fatty acid double bonds (unmodified LO, Supplementary Material, Figure S1a) disappear, as a result of the epoxidation [17,19,20]. FTIR analysis (Supplementary Material, Figure S2) confirms the epoxidized structure of the LO monomer, through the appearance of the C–O–C stretching vibration band (epoxy rings) at 825 cm^−1^. The absorptions bands at 3010 and 1651 cm^−1^ associated to sp2 C–H and to C=C stretching vibrations, respectively, are here relatively weak [17,19,20].

### 3.2. ELO-LnK Composite Synthesis and Characterization

Diamines and anhydrides were tentatively used as crosslinkers for ELO. In presence of LnK, diamines like hexamethylenediamine (HAD) failed to lead to proper composites. The unsuccessful process can be associated to competing reaction of the functional groups from the lignin structure with the amino groups of HAD leading to polycondensation products, according to literature reports [16,24], resulting in LnK sedimentation and poor crosslinked ELO network. In the absence of LnK we were able to use diamine as crosslinking agent for ELO.

In another approach, cationic photocuring of the ELO-LnK system was performed using THA as a photo-initiator. However, those systems with low LnK content (5%) did not harden, but formed a thin film on the surface after several UV irradiation cycles (at 365 nm). The UV stabilizing effect exerted by the LnK can be an argue, in accordance with the established feature of the kraft lignin as a filler in UV-curable systems to prevent the quenching phenomenon (fluorescence) [25,26]. Thus, the adopted procedure was a double-curing process using UV irradiation at room temperature for 15 min. (ELO homopolymerization), followed by thermal crosslinking at 80 °C for 23 h.

### 3.3. FTIR Spectrometry

The efficiency of the selected curing procedures was assessed by FTIR spectrometry, in 600–4000 cm^−1^ wave number region, before and after the dual treatment.

**Figure 1:**
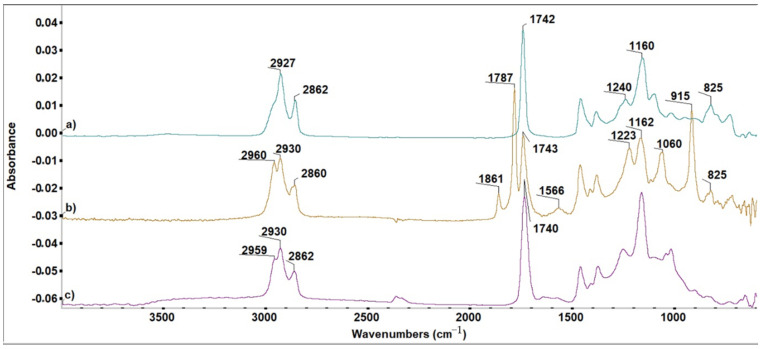
FTIR spectra of (**a**) ELO; (**b**) ELO-based formulation before curing; (**c**) product resulted after curing (Sr).

For our discussion, the FTIR spectrum of ELO (Figure 1a) shows a typical absorption at 825 cm^−1^ attributed to the stretching vibration of epoxy rings. The (b) spectrum (Figure 1b) corresponds to the unreacted mixture ELO-HY-DY-THA, with the expected characteristic bands: 825 cm^−1^ (typical for the epoxy ring), 915 cm^−1^ (δ_C–H, oop_ aromatic folding), 1060 cm^−1^ (δ_C–O_, HY 964 anhydride), 1223 cm^−1^ (δ_C–N,_ DY 064), 1566 cm^−1^ (δ_C=C_), 1787 and 1861 cm^−1^ (δ_C=O_, anhydride). In the (c) spectrum (Figure 1c) of the cured material, the absorption band of the epoxy rings (825 cm^−1^) and those of the other reactants (1060, 1223, 1566, 1787, and 1861 cm^−1^) disappeared, as expected after crosslinking and photopolymerization of ELO. The new bands in the range 1000–1100 cm^−1^ can be ascribed to the new C–O bonds formed during the two curing processes.

**Figure 2:**
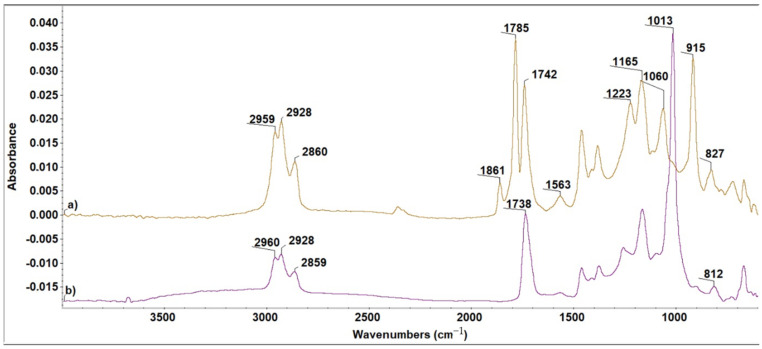
FTIR spectra of the S1 system (ELO + 5%LnK). (**a**) Before and (**b**) after dual-curing reaction.

For the ELO-LnK composites, similar findings were noticed (Figure 2).

The appearance of the high intensity band at 1013 cm^−1^ is due to the new C–O bonds formed during the curing processes. Well defined pick shape can be argued by a great number of C–O bonds coming not only from ELO matrix, but also from the new reaction points between ELO and LnK, as a first indication of a reinforcement effect which can be produced by LnK.

Additionally, for all the studied ELO-based materials, *GF measurements* indicate small unreacted fraction (maximum 8%) after the applied dual-treatment (UV irradiation and thermal curing). A slightly increase (3%) of the GF value was noticed for the S3 system compared to the reference sample (Table 2).

### 3.4. Thermogravimetric Analysis

The thermal stability of the composites obtained by dual-curing treatment was investigated by TGA, in N_2_ atmosphere as well as in presence of air. Results are summarized in Table 1.

All ELO and ELO-LnK composites show in inert atmosphere a similar behavior, indicating a comparable thermal stability. When LnK is loaded, a lower degradation rate was noticed for all samples (S1–S3) as compared to the reference sample Sr, indicating an improved thermal stability associated with the aromatic structure of the filler. For the S2 system, a slight decrease of Td values in the entire temperature range was observed. Such behavior could be explained by a poor LnK dispersion within ELO-based matrix, as a result of an inefficient homogenization. As can be seen from the weight loss curves, above 300 °C, the reference material (Sr) decomposes faster leading to lower residual char at 700 °C (1%), due to the complete decomposition of the LO aliphatic chains. The remaining char quantity is due, probably, to the THA initiator and DY064 accelerator aromatic structures. The increase of residual mass at 700 °C for the S1–S3 composites could be explained by the crosslinked phenolic-type structure of LnK, which does not easily break down [27].

**Figure 3:**
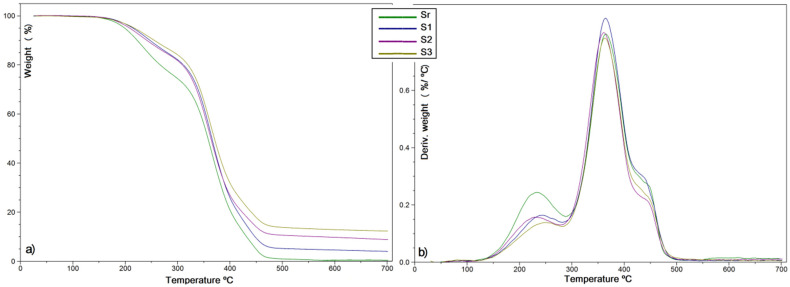
(**a**) TGA and (**b**) DTG curves for ELO-derived composite materials (N_2_).

All DTG curves showed a three-step decomposition behavior (Figure 3b). The weight loss onset, up to 300 °C (representing ~20% of sample weight) could be attributed to the volatilization of decomposition products with low molecular weight and low boiling point. The next mass loss stage (of ~65%), with the maximum rate around 365 °C could be attributed to the decomposition of polymeric and oligomeric fragments, whereas the last weight loss (of ~15%), transposed in the DTG curves as a shoulder around 450 °C, probably corresponds to C–C bond cleavage [28].

**Figure 4:**
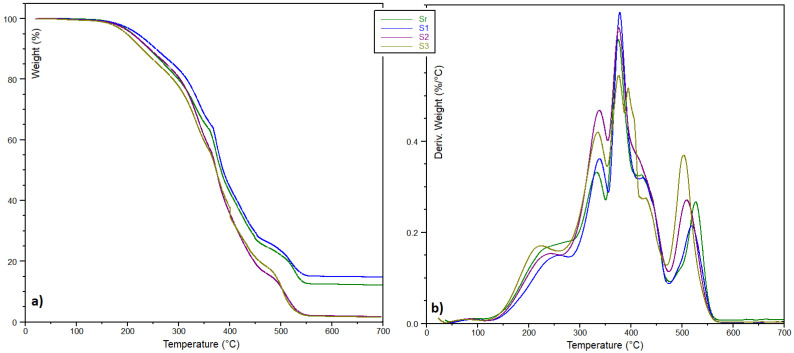
Thermal degradation profile (air) of ELO-LnK composites. (**a**) TGA and (**b**) DTG curves.

TGA studies of the ELO-LnK composites in the presence of air showed a more complex decomposition process (Table 1). Curve shapes are slightly dependent on the LnK proportion in the composite (Figure 4), the decomposition process under thermo-oxidative condition being faster once the concentration of the LnK increases. ELO-based system with lower LnK amount (S1, 5% wt. LnK) seems to be the more thermo-stable material in oxidative environment.

The degradation behavior (Figure 4b) could be related to the LnK amount, but also to the heterogeneity of the triglyceride structure, which means a wider distribution of molecular weight segments within the cross-linked materials.

Decomposition broad peaks, at 150–280 °C (T_max_1), are noted for all the samples, which can be explained by moisture elimination but also by loss of volatile compounds and low molecular fragments. At elevated temperatures, two well-defined exothermal peaks are observed (280–450 °C), with shoulder-like peaks around 420–430 °C. These distinct peaks can be explained by degradation processes involving ELO intra-molecular bonds as well as the ELO-LnK inter-unit linkages. When raw LnK was analyzed under the same thermal conditions, a single decomposition peak at 448 °C was registered. Thus, T_max_2 may be attributed to the fragmentation of the ELO matrix, consisting of more flexible chains, which are easily exposed to oxygen attack.

An influence of LnK upon thermo-oxidation processes is also clearly observed, this stage involving probably degradation of LnK substituents. In case of S3 (15% wt. LnK content), two peaks can be observed at 376 and 395 °C, respectively, and a small shoulder at 429 °C. Such a decomposition profile could be associated with a 3D network with multiple bond types between the oil matrix and LnK as well as with different pyrolysis mechanisms involving LnK aromatic rings substituents, expected to be relatively stable in the first pyrolysis stage [29]. Above 500 °C, a last decomposition peak is observed on the DTG graphs, associated with the ELO matrix, with long tails beyond 570 °C.

### 3.5. Dynamic-Mechanical Analysis

Thermo-mechanical properties of the composites were investigated (Table 2). LnK loading significantly shifts the peaks of tan δ, an increase of 20 °C of the tan δ being observed for the S3 composite, as compared with the neat polymer (Sr). This could be strong evidence of the reinforcing effect involving the aromatic structures. Such interactions could restrict the motion of the long fatty acid chains and thus impart stiffness. We may assume that there are fewer dangling chains available in the network, together with lower molecular weight segments (probably located between crosslinking centers).

### 3.6. Morphology Investigation

**Figure 5:**
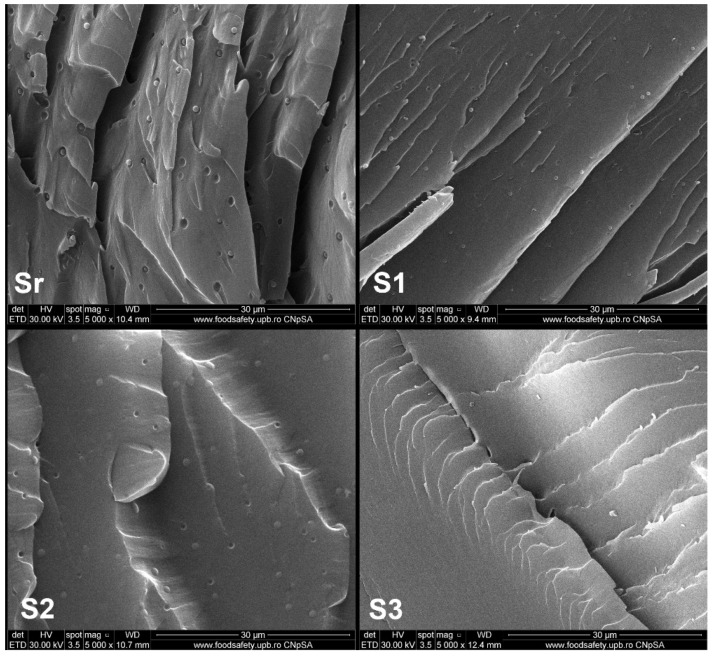
Cross-section SEM images of ELO-LnK composites (5000× magnification). Sr: ELO polymer; S1: ELO+5% wt. LnK; S2: ELO+10% wt. LnK; S3: ELO+15% wt. LnK.

Figure 5 shows SEM photographs of the fractured neat ELO material and respectively ELO-LnK composites S1–S3, at 5000× magnification. It can be observed a single-phase morphology for all the investigated specimens, with certain architectural differences associated with the LnK content. The areas of fracture seem to undergo modifications with increasing LnK content.

The fracture surface of the neat epoxy polymer (Figure 5, Sr) is very smooth except for the regular button-shape observed in the cross section, which comes from the incorporated THA photo-initiator. This attribution has been proven by SEM-EDX analysis (Supplementary Material—Figure S4). In contrast, the fracture surfaces of ELO-LnK materials seem rougher (Figure 5 S1–S3). S1 and S3 samples exhibit a network of fine cracks. For S3, we also noticed the disappearance of the button-shape coming from THA.

### 3.7. Water Affinity

**Figure 6:**
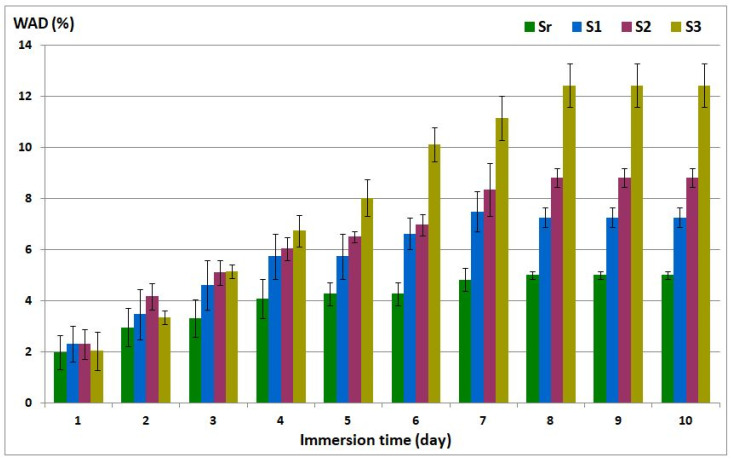
Water absorption degree for ELO-LnK composites.

Static water contact angle measurements (CA) and water absorption experiments (WAD) were carried out to evaluate the wettability of the composites. The results are shown in Table 2 and Figure 6, respectively.

Generally, water affinity has a direct effect on corrosion susceptibility, more hydrophobic surfaces conferring enhanced resistance against wet corrosion [30].

Lignin, which has both hydrophilic and hydrophobic units, may influence the wetting properties of the final materials in both directions. A highly hydrophobic polymer matrix, as provided by ELO, is expected to reduce the hydrophilic character of the materials, improving at the same time their stability towards thermal degradation and towards corrosive media. CA values can thus be naturally influenced by the LnK concentration and dispersion, the latter influencing the wetting behavior of a surface.

The sample with 15% wt. LnK concentration (S3) registered the lowest contact angle value, around 71°, the differences between the neat Sr sample and S1 (containing 5% wt. LnK) being just 8°. If LnK-loaded composites are compared with the reference sample, the decrease of the CA values is not too high considering the large number of –OH groups (phenolic and aliphatic) of the LnK structure.

To elucidate the bulk water affinity of the ELO-LnK composites, water absorption degree (WAD) was investigated for ten days, using a ASTM D570 method.

The WAD graph (Figure 6) reveals a low water affinity after ten days of immersion: 7.26% for S1, 8.83% for S2, and 12.42% for S3. These values are promising for the corrosion protection application when compared with other lignin-epoxy composites as anticorrosion coating [31].

### 3.8. Electrochemical Measurements

**Figure 7:**
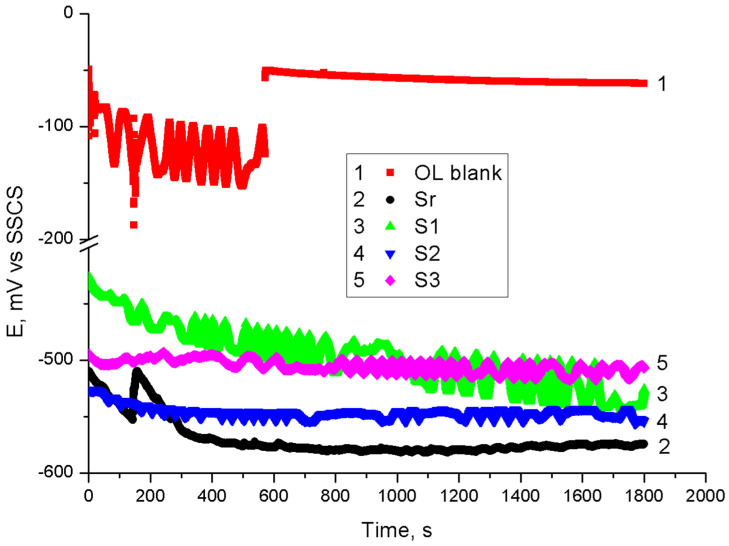
OCP time curves for coated samples Sr (2), S1 (3), S2 (4), and S3 (5).

The anticorrosion performance of ELO-LnK composites was evaluated using coated carbon steel sheets. Figure 7 shows the open circuit potential (OCP) curves vs. time obtained for samples coated with ELO-LnK (Sr, S1, S2, S3) in 3.5% NaCl solution. In the absence of LnK, the initial potential is shifted towards more negative values (curve 2), showing for a short time a shift towards more positive values, maybe due to the porosity of the coating and formation of an oxide film. OCP stabilized gradually with prolonged immersion time up to 600 s. The addition of the LnK leads to a shift of the potential in open circuit towards more positive values. The samples with higher LnK content (S2 and S3–curves 4 and 5) reach a stable potential value shortly after immersion (in about 200 s), while S1 (curve 3) shows a higher potential instability, which might be related to a higher porosity.

**Figure 8:**
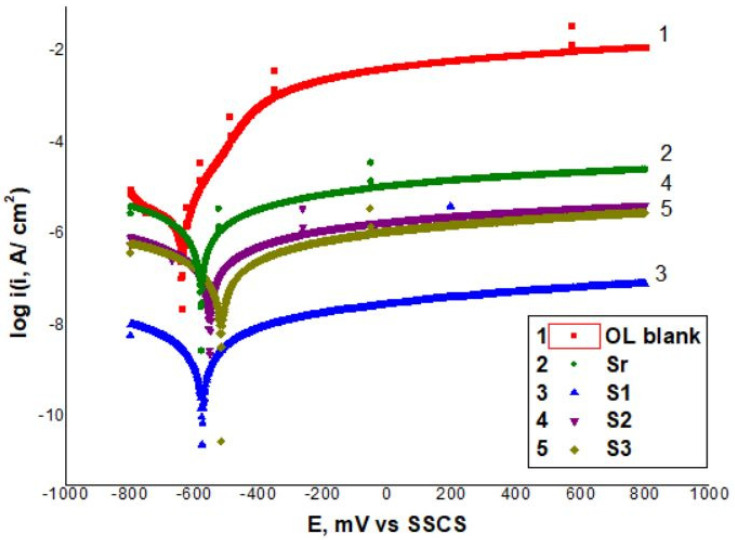
Potentiodynamic polarization (Tafel) curves for uncoated and coated samples in 3.5% NaCl solution at 25 °C (OL—the uncoated sheet/blank sample).

Potentiodynamic polarizations experiments in 3.5% NaCl solution for 30 min (Figure 8) were conducted to measure parameters such as corrosion potential (E_corr_), corrosion current density (*i*_corr_), polarization resistance (R_p_) for the carbon steel sheets coated with ELO-LnK layers. Table 3 shows these results together with cathodic (βc) and anodic (βa) Tafel slopes and inhibition efficiency (*IE*).

The corrosion inhibition efficiency (IE) was calculated using Equation (2):

(2) $$\mathrm{IE}\;=\;\left(1-\frac{i_{\mathrm{corr}}}{i_{corr}^{0}}\right)\times 100\%$$

where $i_{corr}^{0}$ and $i_{\mathrm{corr}}$ are the current densities for steel corrosion in the absence and respectively in the presence of ELO-based coating, in 3.5% NaCl solution.

The graphs in Figure 8 show clearly that all ELO-based coatings exhibit a corrosion inhibition effect, significantly reducing the density of the corrosion current. With or without LnK the coatings lead to a faster passivation of steel surface. With 5% LnK (S1 sample), the corrosion rate is reduced by up to two orders of magnitude compared with the uncoated sample (OL). The highest efficiency against corrosion, namely 99.9%, is exhibited by the S1 sample, while the lowest, 64.19%, by Sr which contains no LnK.

**Figure 9:**
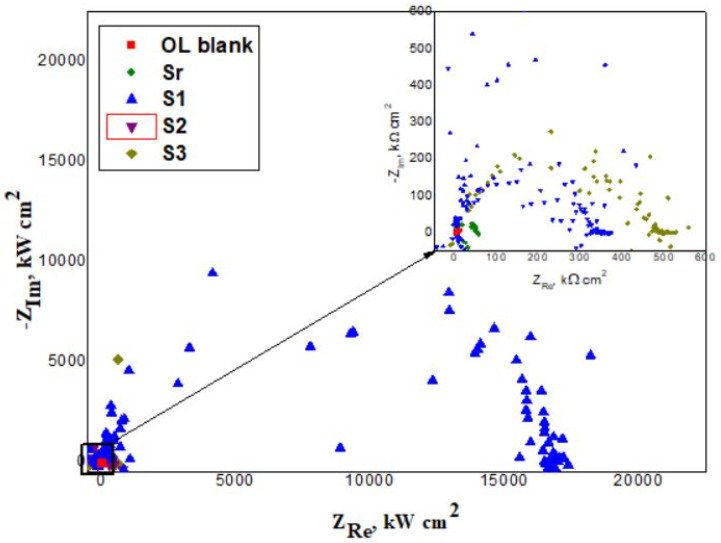
Nyquist plots after accelerated corrosion test in 3.5% NaCl solution at 25 °C.

Electrochemical impedance spectroscopy (EIS) measurements were performed on the coated steel samples. Nyquist plots were registered before the potentiodynamic polarization test in aggressive environment (after 30 min immersion) as well as after the test (after accelerated corrosion test) (Figure 9). Electrochemical parameters such as electrolyte resistance (R1), coating resistance (R2), double layer capacitance (Cdl), are presented in Table 4. The inhibition efficiency (IE) was calculated using Equation (3):

(3) $$\mathrm{IE}=\left(1-\frac{R_{ct}^{0}}{R_{\mathrm{ct}}}\right)\times 100\%$$

where $R_{\mathrm{ct}}$ and $R_{ct}^{0}$ are the charge transfer resistance for the coated samples and respectively for the uncoated one. The corresponding results are shown in Table 4.

As shown in Table 4, after the 30 min immersion required to stabilize the system in the aggressive environment, LnK-loaded coatings indicated a superior corrosion performance, S1 (5% LnK) being the more efficient. The results after accelerated corrosion test are found to be comparable with those obtained after 30 min immersion (Table 4).

The Nyquist recorded spectra (Figure 9), show a semicircle, the higher diameter being associated with the S1 sample (5% LnK). These results are in good agreement with those obtained from Tafel measurements.

## 4. Conclusions

Considering the global issues regarding the extensive use of petroleum-based raw materials that generate non-biodegradable products, here were formulated, obtained, and tested new composite materials based on renewable resources: vegetable oil and lignin, as anticorrosion protective layers for carbon steel.

The obtained bio-based epoxy resin derived from linseed oil was used further to produce the new composites by loading lignin in different mass ratios. FTIR spectrometry and GF measurements indicated the success of the double-curing procedure involving UV irradiation and thermal treatment, respectively. An improved dispersion of the LnK into the polymer matrix was attained by the double-curing technique. SEM images indicate the good dispersibility of the lignin particle within oil-based network. We intend in a future project to systematically investigate this dual-curing procedure and the polymer thus resulted.

Thermal degradation of the bio-based materials was investigated by TGA in both inert and air-oxidant atmosphere registering higher stability for all the LnK-loaded samples in N2, and an improved stability for the sample with 5% wt LnK in aerobic condition. Water affinity was not dramatically influenced when LnK was added, but the thermo-mechanical features were improved indicating the reinforcement effect generated by the rigid aromatic units from the lignin structure.

ELO-LnK composites showed promising anticorrosion protection performance. The sample with 5%-LnK displayed the highest corrosion inhibition efficiency both in Tafel polarization curves and in Nyquist plots.

## Figures and Tables

**Table 1:** TGA data of ELO and LnK-based products (N_2_ and air).

N_2_ Atmosphere											
**Sample** **Code**	**Weight Loss**				**Degradation Steps**						**Weight Change at 700 °C (%)**
	**T_d_^a^**				**T_max_^b^**						
	**3% (°C)**	**10% (°** **C)**	**30% (°C)**	**50% (°C)**	**1 (°** **C)**		**2 (°** **C)**		**3 (°** **C)**		
Sr	185	223	320	358	234		365		448 s		99
S1	197	247	339	366	243		365		440 s		96
S2	192	242	336	364	230		362		448 s		91
S3	197	256	343	370	250		363		449 s		88
LnK	159	258	355	502	-		353		-		61
**Air Atmosphere**											
**Sample** **Code**	**Weight Loss**				**Degradation Steps**						**Weight Change at 700 °C (%)**
	**T_d_^a^**				**T_max_^b^**						
	**3% (°C)**	**10% (°** **C)**	**30% (°C)**	**50% (°C)**	**1 (°** **C)**	**2 (°** **C)**		**3 (°** **C)**		**4 (°** **C)**	
Sr	193	242	336	383	230	333		375 421 s		527	88
S1	199	256	346	387	260	339		378 426 s		520	85
S2	191	243	332	374	243	338		376 417 s		509	98
S3	182	229	325	373	224	336		376 395 429 s		503	98
LnK	86	255	395	436	-	-		448		-	93

a—Td = the thermal degradation temperature as the weight loss of material at 3, 10, 30, 50%; b—Tmax = temperature at which the maximum mass decomposition occurs; s—shoulder.

**Table 2:** DMA, GF, and contact angle results for ELO-LnK composites.

Sample	Tg ^c^ (°C)	GF ^d^ (%)	Θ ^e^ (°)
Sr	55	92.06 ± 0.54	84.82 ± 1.72
S1	58	93.13 ± 0.24	77.89 ± 4.07
S2	62	93.95 ± 0.22	73.97 ± 1.46
S3	75	94.99 ± 0.30	71.38 ± 1.95

c—Tg = glass transition temperature considered as the maximum of tan δ plots; d—GF = gel fraction (the average of three measurements and corresponding standard deviation); e—θ = water contact angle (the average of three measurements and corresponding standard deviation).

**Table 3:** Electrochemical parameters calculated from Tafel polarization curves.

Sample Code	E_corr_ (mV/SSCS)	*i_corr_* (µA/cm^2^)	Rp (kohm * cm^2^)	βa (mV)	−βc (mV)	Correlation Coefficient	IE (%)
OL *	−646	1.5531	12.64	75.3	257.3	0.9999	-
Sr	−584	0.5562	52.71	180.8	185.0	0.9977	64.19
S1	−581	0.0015	19,930.00	186.1	188.3	0.9974	99.90
S2	−558	0.1036	282.20	185.1	183.3	0.9977	93.33
S3	−523	0.0601	450.26	171.0	170.6	0.9977	96.13

* OL—the uncoated carbon steel sheets (blank).

**Table 4:** Electrochemical parameters obtained from EIS measurements.

Sample Code	A *				B *			
	R1 (ohm * cm^2^)	R2 (kohm * cm^2^)	Cdl (pF/cm^2^)	IE (%)	R1 (ohm * cm^2^)	R2 (kohm * cm^2^)	Cdl (pF/cm^2^)	IE (%)
OL	111.80	6.48	43.69 10^6^	-	45.83	37.50	84.87 10^6^	-
Sr	63.83	41.43	307.30	84.35	38.52	49.53	321.20	24.29
S1	92.99	20,600.00	691.90	99.97	41.63	17,350.00	366.80	99.78
S2	132.00	405.90	43.90	98.40	224.00	596.30	53.37	93.71
S3	70.04	390.40	290.10	98.34	40.90	501.70	225.80	92.53

A: after 30 min immersion in the 3.5% NaCl solution; B: after the accelerated corrosion test in the 3.5% NaCl solution.

## Data Availability

The data presented in this paper are available on request from the corresponding author.

## Supplementary Materials

The following are available online at https://www.mdpi.com/article/10.3390/polym13213792/s1, Figure S1: ^1^H-NMR spectra of (a) crude LO and (b) epoxidized LO; Figure S2—FTIR spectra of crude LO (a) and epoxidized LO (b); Figure S3—Tan delta versus temperature registered for the ELO-LnK materials; Figure S4—Qualitative SEM-EDX for Sr sample (SEM at 1000× magnitude). Insert: quantitative results for ELO-based composites.
