# Supplementary material for: Composite Materials from Renewable Resources as Sustainable Corrosion Protection Coatings
Source: Polymers (Basel). 2021 Nov 2;13(21):3792. doi: 10.3390/polym13213792 (PMC8588247; doi:10.3390/polym13213792)
Supplement: Supplementary file 1 [file polymers-13-03792-s001.zip › Polym1439441-SM.pdf]

Supllementary Materials

Composite Materials from Renewable Resources as Sustainable Corrosion Protection Coatings

Polymers **2021**, *13*(21), 3792; https://doi.org/10.3390/polym13213792

Supllementary Materials

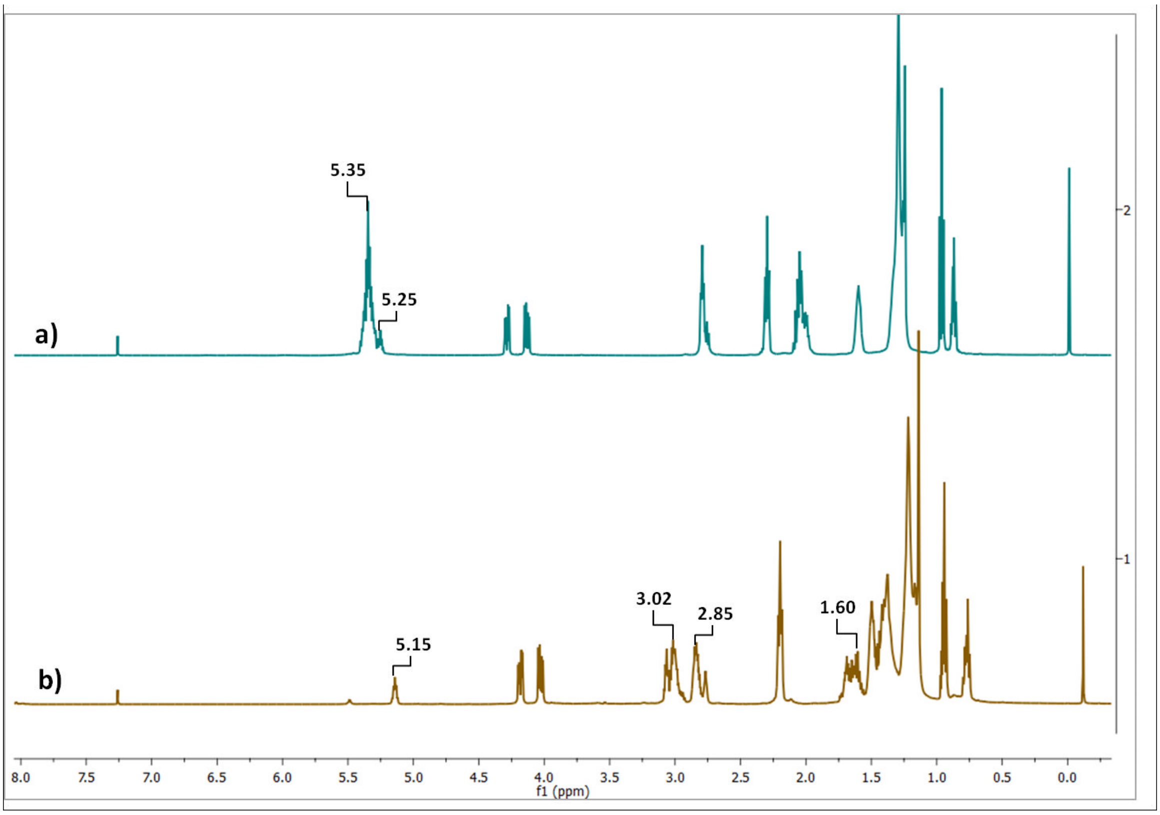

Figure S1 – ^{1}H-NMR spectra of a) crude LO and b) epoxidized LO

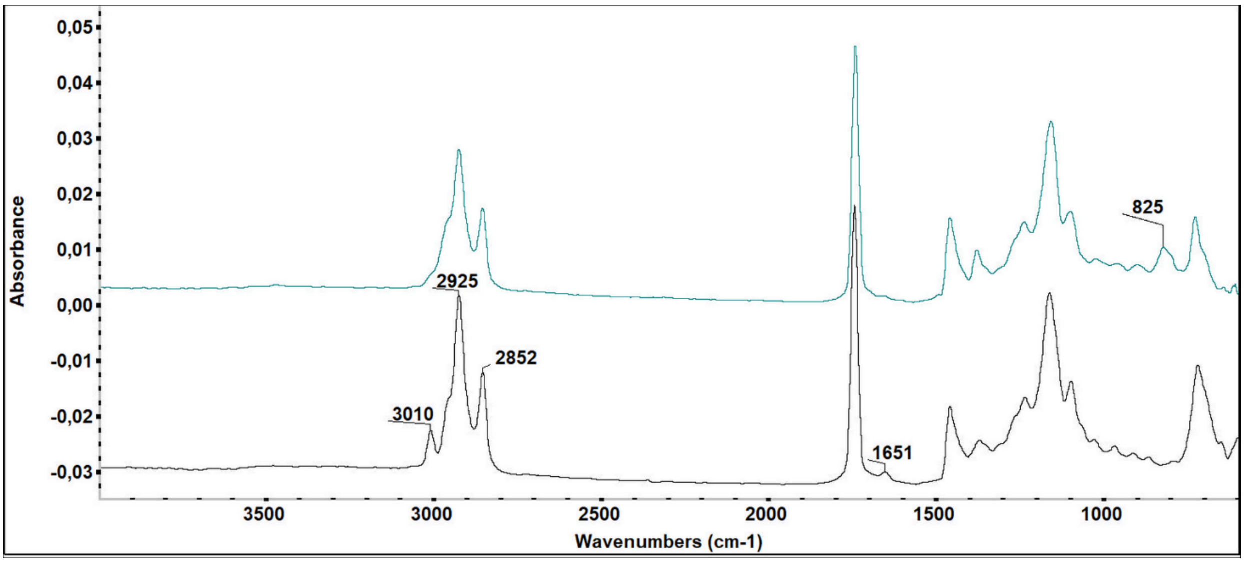

Figure S2 – FTIR spectra of crude LO (a) and epoxidized LO (b)

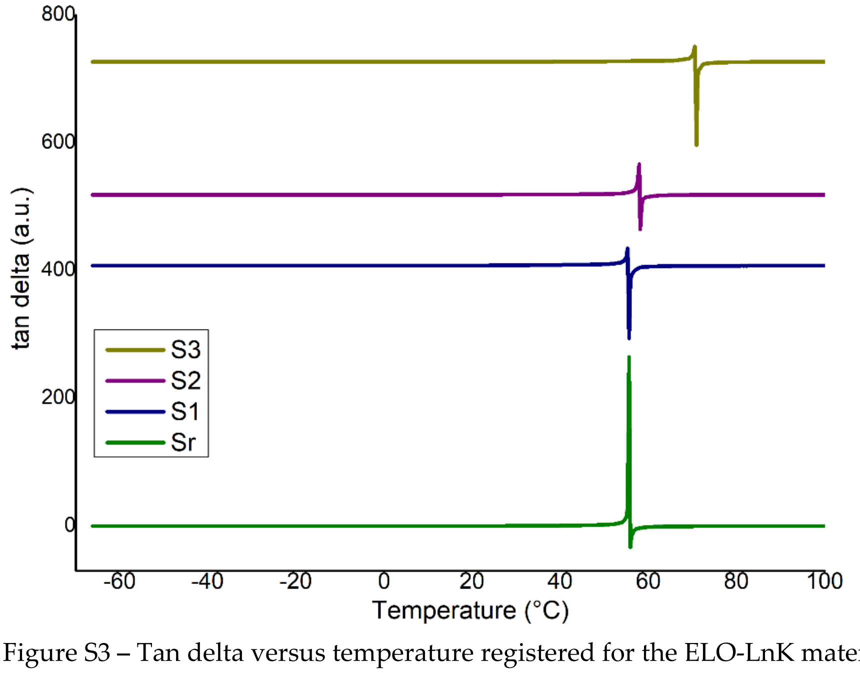

Figure S3 – Tan delta versus temperature registered for the ELO-LnK materials

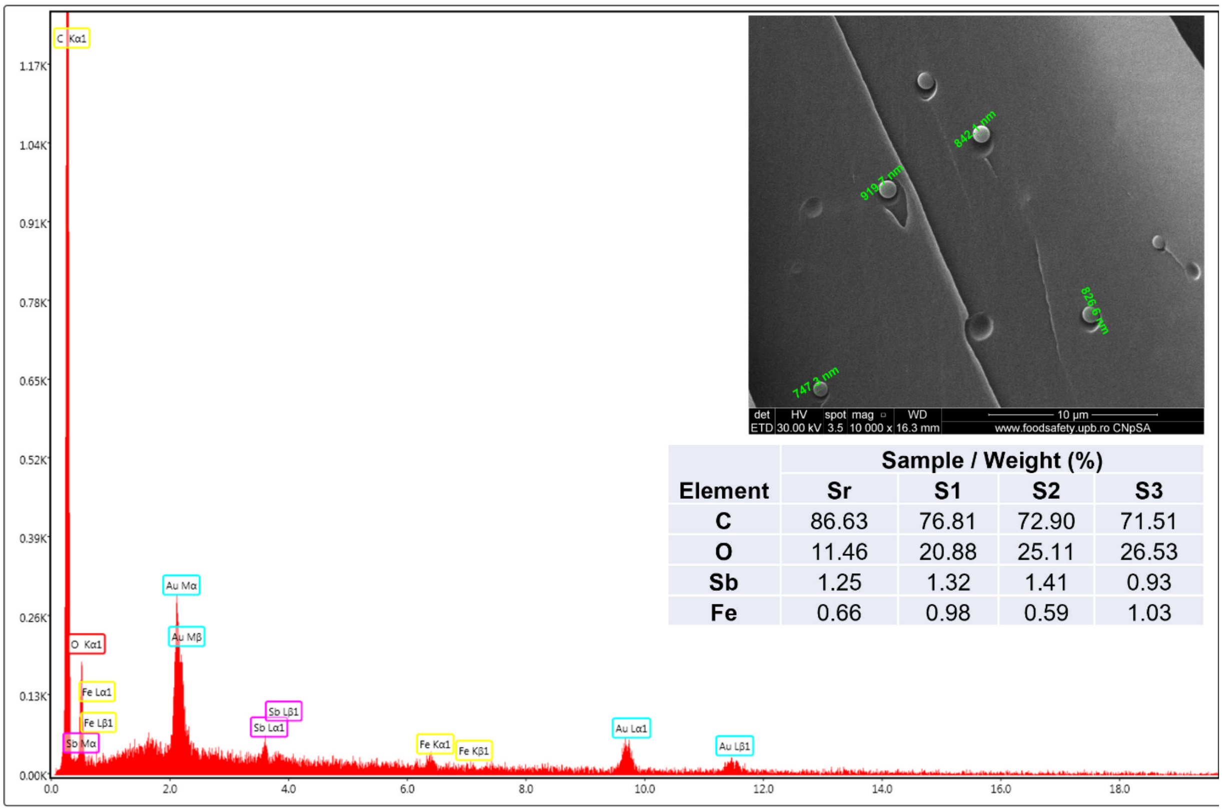

Element	Sample / Weight (%)			
	Sr	S1	S2	S3
C	86.63	76.81	72.90	71.51
O	11.46	20.88	25.11	26.53
Sb	1.25	1.32	1.41	0.93
Fe	0.66	0.98	0.59	1.03

Figure S4 – Qualitative SEM-EDX for Sr sample (SEM at 10000x magnitude). Insert: quantitative results for ELO-based composites
